# Fashionable Freaks

**Published:** 1885-11

**Authors:** 


### Fashionable Freaks.

When the French nation reached its height of folly and wickedness, just before the Revolution broke out and flooded the land with misery and bloodshed, all who desired to be considered connected with the aristocracy carried about with them at least one *pantin.* These were small wooden dolls which, by pulling a string, suddenly jerked out arms and legs, exactly like those which may be seen adorning the hats of "swells" on a Derby day. The rage for them was immense. Nobles, gentlemen, and even grave ecclesiastics were to be seen carrying them about and playing with them. A somewhat similar rage for comfits existed in the reign of Henry III. of France. When the body of the Duc de Guise was found after the battle of Blois he had a comfit-box in his hand. In 1585 the ladies carried hand mirrors attached to their chatelains, and like Narcissus were perpetually admiring their own charms. This excited the deepest indignation of Jean des Causes, a stern old moralist of the time, and he emphatically menaced them with the extremest penalties of the other world Who would have believed that so late as 1751 the dress of a dandy should have consisted of a black velvet coat, a green and silver waistcoat, yellow velvet breeches and blue stockings! A satirical writer of about the same period gives a biting sketch of one of his contemporaries: "A coat of light green, with sleeves too small for the arms, and buttons too big for the sleeves; a pair of Manchester fine-stuff breeches, without any money in the pockets; clouded silk stockings, but no legs; a club of hair behind larger than the head that carries it; a hat of the size of a sixpence on a block not worth a farthing." No doubt the same gentleman could paint a picture of the dress of our own time which would appear as ridiculous to the gentleman with the green coat as his own does to us.

